# Study of indications for cardiac device implantation and utilisation in Fabry cardiomyopathy

**DOI:** 10.1136/heartjnl-2019-315229

**Published:** 2019-08-24

**Authors:** Ravi Vijapurapu, Tarekegn Geberhiwot, Ana Jovanovic, Shanat Baig, Sabrina Nordin, Rebecca Kozor, Francisco Leyva, Dipak Kotecha, Nigel Wheeldon, Patrick Deegan, Rosemary A Rusk, James C Moon, Derralynn A Hughes, Peter Woolfson, Richard P Steeds

**Affiliations:** 1 Department of Cardiology, Queen Elizabeth Hospital Birmingham, Birmingham, UK; 2 Institute of Cardiovascular Sciences, University of Birmingham, Birmingham, UK; 3 Department of Endocrinology, Queen Elizabeth Hospital Birmingham, Birmingham, UK; 4 Institute of Metabolism and System Research, University of Birmingham, Birmingham, UK; 5 Mark Holland Metabolic Unit, Salford Royal Hospitals NHS Trust, Salford, UK; 6 Department of Cardiology, Barts Heart Centre, London, UK; 7 Sydney Medical School, University of Sydney, Sydney, New South Wales, Australia; 8 Aston Medical Research Institute, Aston Medical School, Birmingham, UK; 9 South Yorkshire Cardiothoracic Centre, Northern General Hospital, Sheffield, UK; 10 Department of Medicine, Cambridge University Hospitals NHS Foundation Trust, Cambridge, UK; 11 Department of Cardiology, Cambridge University Hospitals NHS Foundation Trust, Cambridge, UK; 12 Lysosomal Storage Disorder Unit, Royal Free London NHS Foundation Trust, London, UK; 13 Department of Cardiology, Salford Royal Hospitals NHS Trust, Salford, UK

**Keywords:** fabry, arrhythmia, defibrillator, prognosis, risk

## Abstract

**Background:**

Fabry disease is a treatable X-linked condition leading to progressive cardiomyopathy, arrhythmia and premature death. Atrial and ventricular arrhythmias contribute significantly to adverse prognosis; however, guidance to determine which patients require cardiovascular implantable electronic devices (CIEDs) is sparse. We aimed to evaluate indications for implantation practice in the UK and quantify device utilisation.

**Methods:**

In this retrospective study, we included demographic, clinical and imaging data from patients in four of the largest UK Fabry centres. Ninety patients with Fabry disease were identified with CIEDs implanted between June 2001 and February 2018 (FD-CIED group). To investigate differences in clinical and imaging markers between those with and without devices, these patients were compared with 276 patients without a CIED (FD-control).

**Results:**

In the FD-CIED group, 92% of patients with permanent pacemakers but only 28% with implantable cardioverter-defibrillators had a class 1 indication for implantation. A further 44% of patients had defibrillators inserted for primary prevention outside of current guidance. The burden of arrhythmia requiring treatment in the FD-CIED group was high (asymptomatic atrial fibrillation:

29%; non-sustained ventricular tachycardia requiring medical therapy alone: 26%; sustained ventricular tachycardia needing anti-tachycardia pacing/defibrillation: 28%). Those with devices were older, had greater LV mass, more scar tissue and larger atrial size.

**Conclusions:**

Arrhythmias are common in Fabry patients. Those with cardiac devices had high rates of atrial fibrillation requiring anticoagulation and ventricular arrhythmia needing device treatment. These are as high as those in hypertrophic cardiomyopathy, supporting the need for Fabry-specific indications for device implantation.

## Introduction

Fabry disease (FD) is an X-linked lysosomal storage disorder caused by a deficiency in the enzyme α-Galactosidase A,^1^ which leads to progressive accumulation of sphingolipids.^2^ This leads to cellular dysfunction and life-threatening cardiovascular, renal and neurological complications.^3^ The advent of enzyme replacement (ERT) and oral chaperone therapy has altered the natural history of FD, with cardiovascular disease now the main cause of morbidity and mortality.^4^ Cardiac involvement includes progressive left ventricular hypertrophy (LVH), myocardial inflammation, fibrosis, arrhythmia, congestive cardiac failure and sudden death.^5^ Sphingolipids accumulate in all cardiac cells including the conduction system.^6^ This triggers a cascade of cellular reactions leading to a proinflammatory microenvironment with local tissue injury and apoptosis.^7^ The ensuing damage to conductive tissue contributes to electrical instability and subsequent development of arrhythmia. Although symptoms such as palpitations and syncope are common in FD, little is known regarding the true frequency of arrhythmia.^4^ Registry data and small single centre studies suggest that the rate of atrial arrhythmias such as atrial fibrillation (AF) could be as high as 13%,^8^ while the reported incidence of ventricular arrhythmia varies widely from 5% to 30%,^9–11^ with a progressive increase with advancing age.^12^

A number of clinical and imaging parameters have been identified as markers of potential arrhythmia,^13 14^ but no criteria exist to guide implantation of cardiovascular implantable electronic devices (CIEDs) for primary prevention. Furthermore, FD is specifically excluded from the risk prediction tool for sudden cardiac death used in hypertrophic cardiomyopathy (HCM),^15^ despite similarities in risk factors between FD and HCM.^16^ Data are lacking on the reasons for implantation of CIEDs in FD and on device utilisation following implantation.^17 18^

The aims of our study were to evaluate the indications for CIEDs applied in clinical practice, to quantify arrhythmia burden and device usage in patients with FD and to investigate differences in clinical characteristics between those with and without a device.

## Methods

### Study population

This study conformed to the principles of Good Clinical Practice guidelines. The study consisted of patients with genetically confirmed FD followed up at national specialist centres within the UK: Queen Elizabeth Hospital, Birmingham; Salford Royal Hospital, Salford; Royal Free Hospital, London; and Addenbrookes Hospital, Cambridge. We included all patients who had a therapeutic CIED, including a permanent pacemaker (PPM), implantable cardioverter-defibrillator (ICD) or cardiac resynchronisation therapy (CRT), implanted between 1 June 2001 and 1 February 2018 (FD-CIED group). The clinical notes of all patients both current and historical under follow-up within each centre were manually reviewed to identify whether a cardiac device had been implanted and to define the study cohort. A comparator group included patients recruited to the Fabry400 study (ClinicalTrials.gov: NCT03199001), which is a separate observational study evaluating the role of cardiovascular magnetic resonance (CMR) imaging in FD.^19^

### Baseline assessment

Baseline FD specific information included genetic mutation (classical or non-classical variant), other FD-target organ involvement and the Mainz Severity Score Index (MSSI).^20^ The presence of ischaemic heart disease (IHD) was defined as evidence of a flow-limiting lesion on coronary angiography requiring treatment (surgical, percutaneous or medical). In the FD-CIED group, cardiac investigations were recorded if these had been performed before or within 3 months following device implantation. These included transthoracic echocardiography, CMR imaging and a 12-lead electrocardiogram (ECG), which were performed in accordance with standardised protocols. In the FD-control cohort, the most recent investigations during the Fabry400 study period were captured through clinical record analysis. Classification of an ECG as abnormal included the presence of the following: prolonged or shortened PR interval, QRS duration >120 ms, minor conduction disturbances (intraventricular conduction abnormalities and bundle branch block patterns <120 ms), the presence of LVH by Sokolow-Lyon criteria, T wave inversion in at least two contiguous leads and the presence of multifocal ventricular ectopy. LVH was defined as a maximum wall thickness greater than 12 mm, and left atrial dilatation was classified as a biplanar volume indexed to body surface area greater than 28 mL/m^2^.^21^

### Follow-up

CIED follow-up reports were obtained from the date of device implantation until the study end date. If diagnosis of an arrhythmia was unclear from clinical notes, device electrocardiograms were reviewed. Details of changes in treatment were obtained from clinical notes. Table 1 describes the diagnostic criteria for arrhythmic events.

**Table 1:** All arrhythmic events and criteria required for classification

Arrhythmia type	Criteria
Atrial fibrillation	Episode >30 s in duration.
Non-sustained VT	Three or more ventricular beats at a rate >120 bpm for a duration of <30 s.
Sustained VT	Ventricular tachycardia for a duration of >30 s.
VT with haemodynamic compromise	Sustained VT with haemodynamic instability, for example, hypotension or syncope.

VT, ventricular tachycardia; bpm, beats per minute.

### Statistical analysis

Statistical analyses were carried out using SPSS V.23. All continuous variables are expressed as mean±SD, and all non-continuous data are expressed as frequencies or percentages. Normality was evaluated using the Shapiro-Wilk test. Groups were compared with independent t-testing for parametric data and Mann-Whitney U testing for non-parametric data. χ^2^ or Fisher’s exact testing was used to compare proportions within two independent groups. Comparisons between multiple groups were performed using analysis of variance testing with post hoc Tukey correction. Time-to-event analysis was performed to evaluate the presence of arrhythmic events. Kaplan-Meier curves were used to show time to first new diagnosis of AF, ventricular arrhythmia and first appropriate ICD therapy, whereas a multivariable Cox model was used for the occurrence of any arrhythmia requiring treatment during follow-up (online supplementary table 1). Proportionality of hazards was assessed by visual inspection of Kaplan-Meier curves for each predictor variable. A p value of <0.05 was considered statistically significant.

10.1136/heartjnl-2019-315229.supp1 Supplementary data

## Results

### Study cohort characteristics

Of the UK population of 880 Fabry patients in the four national centres, 90 (10%) had a therapeutic cardiac device implanted between 1 June 2001 and 1 February 2018. The FD-CIED group had a median follow-up of 4.3 years (IQR 2.2–7.7 years). These patients are compared with detailed cardiac data from 276 Fabry patients without a device in the FD-control group. Baseline characteristics are described in table 2. The FD-CIED cohort was older, more often male and more frequently had a non-classical mutation (predominantly with a cardiac variant, N215S protein sequence change). Furthermore, disease severity was more advanced in the FD-CIED patients, reflected by higher mean MSSI score, increased prevalence of LVH, ECG abnormalities and late gadolinium enhancement (LGE) on CMR (see table 2). Eighteen patients (20%) in the FD-CIED group had chronic kidney disease stage 3 or above, with two of these patients having undergone renal transplantation, compared with two patients (0.7%) in the controls. Sixty-two patients (69%) were on ERT at time of device implantation, and this was the case in only 131 of the control patients (47%). Baseline Holter monitoring was performed in 52/90 patients (58%) in the FD-CIED group and 37 of these (71%) were abnormal leading to initiation of medical therapy or device implantation (non-sustained ventricular tachycardia (NSVT): 22/52, 42%; SVT: 6/52, 12%; atrioventricular (AV) conduction abnormality: 9/52, 17%). Of note, 19 patients underwent implantation of diagnostic implantable loop recorder (ILR) devices and complete data can be seen in online supplementary table 2.

**Table 2:** Baseline demographic and investigation data: FD-CIED versus FD-control

	FD-CIED	FD-control	P value*
Sample size (n)	90	276	N/A
Age (years)	56±13	46±16	<0.001
Male gender (n, %)	69 (76.7)	100 (36.2)	<0.001
On ERT (n, %)	62 (68.9)	131 (47.5)	<0.001
Classical mutation (n, %)	39 (43)	163 (59)	0.010
BMI (kg/m^2^)	27±6	25±5	0.002
HR (bpm)	71±18	65±13	0.064
SBP (mm Hg)	125±18	123±18	0.406
DBP (mm Hg)	74±11	74±10	0.914
MSSI	15.1±9.7	7.3±7.7	<0.001
Comorbidities			*** ***
IHD (n, %)	6 (6.7)	7 (2.5)	0.115
CKD stage 3–5 (n, %)	18 (20)	26 (9.4)	0.017
eGFR (median, IQR)	82 (46 to 74)	84 (72–90)	0.017
HTN (n, %)	18 (20)	35 (12.7)	0.120
DM (n, %)	8 (8.9)	8 (2.9)	0.046
Stroke/TIA (n, %)	22 (24.4)	11 (4.0)	<0.001
ECG	n=76	n=239	*** ***
Abnormal (n, %)	74 (97.4)	115 (48.1)	<0.001
AF/PAF (n, %)	8 (10.5)	9 (3.7)	0.037
PR interval (ms)	174±40	147±28	<0.001
QRS duration (ms)	136±32	99±20	<0.001
Holter monitoring	n=52	n=85	*** ***
Abnormal	37 (71.2)	12 (14.1)	<0.001
Echocardiography	n=82	n=91	*** ***
LVEF (%)	57±13	62±7	0.002
LVH (n, %)	78 (95.1)	36 (39.6)	<0.001
LA dilatation (n, %)	56 (68.3)	13 (14.3)	<0.001
CMR	n=46	n=210	*** ***
LVMi (g/m^2^)	150±37.3	83±36	<0.001
MWT (mm)	20±4.9	12±4.6	<0.001
LVEDVi (mL/m^2^)	82±32	70±14	0.143
LVESVi (mL/m^2^)	31±31	19±7	0.150
LGE (n, %)	29 (63.0)	85 (40.5)	*<* *0.05*

*P-values are comparing FD-CIED versus FD-control.

AF, atrial fibrillation; BMI, body mass index; CKD, chronic kidney disease; CMR, cardiac magnetic resonance; DBP, diastolic blood pressure; DM, diabetes mellitus; ECG, electrocardiogram; ERT, enzyme replacement therapy; FD-CIED, Fabry disease with cardiovascular implantable electronic device; HR, heart rate; HTN, hypertension; IHD, ischemic heart disease; LA, left atrium; LGE, late gadolinium enhancement; LVEDVi, indexed left ventricular end-diastolic volume; LVEF, left ventricular ejection fraction; LVESVi, indexed left ventricular end-systolic volume; LVH, left ventricular hypertrophy; LVMi, indexed left ventricular mass; MSSI, Mainz Severity Score Index; MWT, maximum wall thickness; PAF, paroxysmal atrial fibrillation; SBP, systolic blood pressure; TIA, transient ischemic attack; eGFR, estimated glomerular filtration rate.

### Cardiac device implantation (FD-CIED group)

The following devices were implanted: PPMs for bradycardia: 38/90 (42%), ICDs for tachyarrhythmia: 43/90 (48%) and CRT for heart failure: 9/90 (10%). Indications for CIED implantation can be seen in table 3. Of those with PPMs, 79% had a class 1 indication for device implantation and 13% a class 2a indication, according to European Society of Cardiology guidance.^17^ The indications for ICD implantation were more variable with only 28% having a class 1 indication for CIED insertion.^18^ Forty-four per cent of patients underwent ICD implantation for primary prevention outside of national guidance (persisting heart failure symptoms with a left ventricular ejection fraction <35% despite optimal medical therapy for 3 months^22^) but based on clinical suspicion of arrhythmia (severe LVH, extensive LGE, abnormal resting ECG, previous NSVT on ECG monitoring or a family history of sudden cardiac death). In all cases, the decision to implant a device followed discussion within an electrophysiology multidisciplinary team meeting. In 26 patients (29%), device implantation preceded a diagnosis of FD. Subgroup analysis of device type showed no differences in baseline demographic data and therefore all therapeutic CIEDs were evaluated as a single cohort (online supplementary table 3).

**Table 3:** Indications for CIED insertion

CIED indication	Frequency	Percentage (%)
PPM (n=38)		
Tachy-brady with coexisting AF	5	13.2
Sinus node dysfunction	9	23.7
Bifascicular and trifascicular block	3	7.9
Second degree AV block	10	26.2
Third degree AV block	9	23.7
No clear indication	2	5.3
ICD (n=43)		
Presumed dual pathology with HCM	7 (three confirmed mutation)	16.3 (7.0)
Symptomatic VT	9	20.9
Multiple risk factors	14	32.6
PPM indication with asymptomatic NSVT	4	9.3
Asymptomatic NSVT	8	18.6
Other (LQTS with syncope)	1	2.3
CRT (n=9)		
Symptomatic LVSD (NYHA class 3) with LBBB	7	77.8
No clear indication	2	22.2

AF, atrial fibrillation; AV, atrioventricular; CIED, cardiovascular implantable electronic device; CRT, cardiac resynchronisation therapy; HCM, hypertrophic cardiomyopathy; ICD, implantable cardioverter-defibrillator; LBBB, left bundle branch block; LQTS, long QT syndrome; LVSD, left ventricular systolic dysfunction; NSVT, non-sustained ventricular tachycardia; NYHA, New York Heart Association; PPM, permanent pacemaker; VT, ventricular tachycardia.

### Follow-up and baseline clinical characteristics

#### Arrhythmia follow-up: FD-CIED group

**Figure 1:**
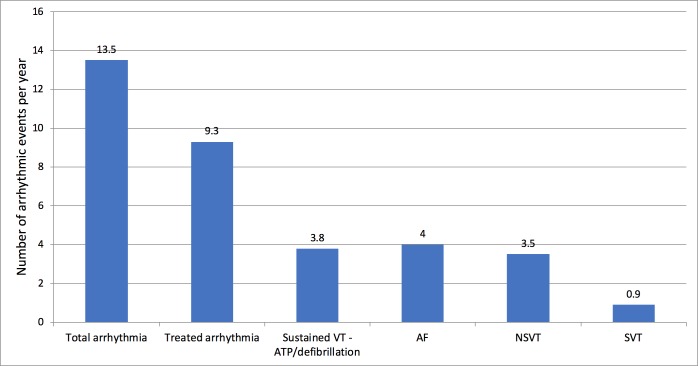
Arrhythmic events per year in the FD cohort with cardiac devices. AF, atrial fibrillation; ATP, antitachycardia pacing; NSVT, non-sustained ventricular tachycardia; SVT, supraventricular tachycardia; VT, ventricular tachycardia.

A total of 58/90 patients (64%) in the FD-CIED group had at least one documented arrhythmia. Forty of these (44%) required initiation of or a change in therapy: 17/58 (29%) were diagnosed with new AF requiring anticoagulation and rate control medication, 4/58 (6.9%) had supraventricular tachycardia (SVT) requiring beta-blocker therapy and 15/58 (26%) had NSVT requiring medical therapy (beta-blocker or amiodarone). Figure 1 demonstrates the incidence of arrhythmic events per year of follow-up. The number of total arrhythmic events per 100 patient years in the FD-CIED group followed a similar trend (table 4).

**Table 4:** Arrhythmic events in the FD-CIED cohort

	Number of patients with at least one event	Events per 100 patient years
All documented arrhythmia	58/90	15.0
Arrhythmia needing treatment	40/90	10.3
AF needing anticoagulation	17/58	6.8
NSVT	15/58	6.0
VT needing ATP and/or defibrillation	14/50	7.6
VT needing defibrillation	8/50	4.3

AF, atrial fibrillation; ATP, antitachycardia pacing; FD-CIED, Fabry disease with a cardiovascular implantable electronic device; NSVT, non-sustained ventricular tachycardia; VT, ventricular tachycardia.

**Figure 2:**
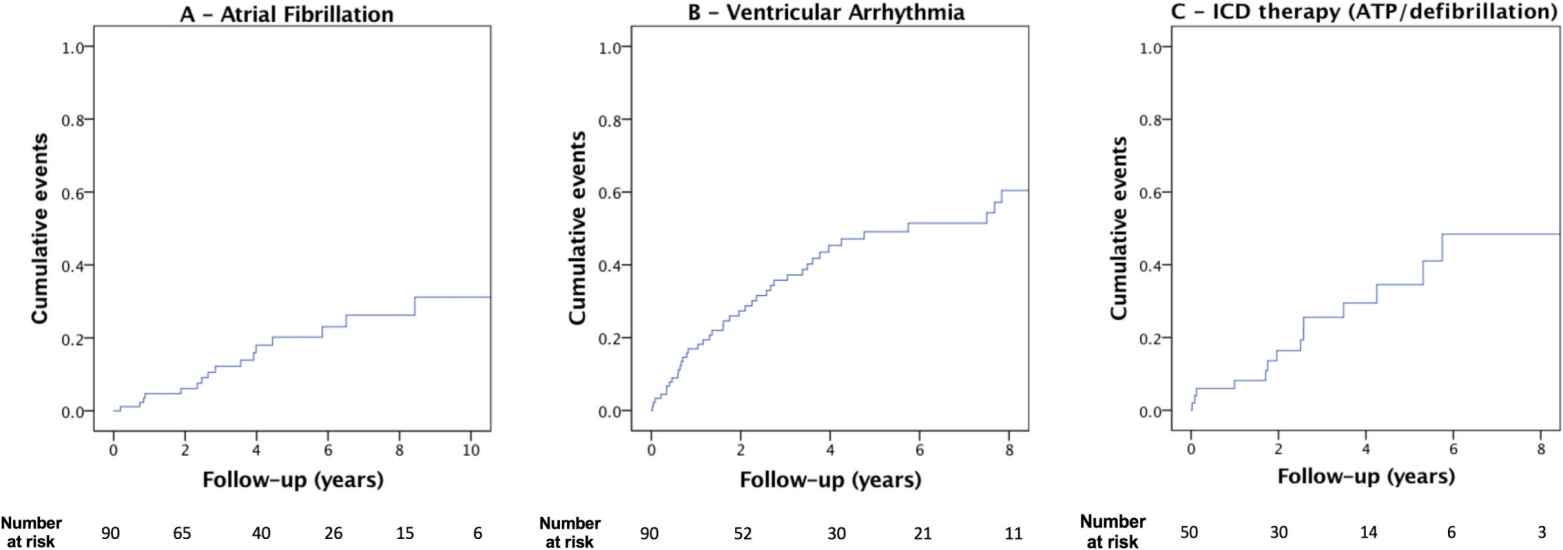
Kaplan-Meier curves illustrating cumulative event rate of atrial and ventricular arrhythmia and cumulative time to first appropriate ICD therapy in the FD cohort with cardiac devices. Panel A: time to first new diagnosis of AF. Panel B: time to first episode of ventricular arrhythmia. Panel C: time to first appropriate ICD therapy (ATP/defibrillation). These Kaplan-Meier curves do not relate to mortality and only the occurrence of an arrhythmic event. AF, atrial fibrillation; ATP, antitachycardia pacing; ICD, implantable cardioverter-defibrillator.

Decision to commence medical therapy for NSVT and subsequent dose titration was variable depending on follow-up centre, frequency of NSVT and the presence of other potential arrhythmic risk factors. Not all patients started on treatment for NSVT were symptomatic. Twenty-five out of 90 (28%) of the FD-CIED cohort had short episodes of asymptomatic NSVT (3–5 beats) during follow-up that were not treated. Time to first atrial and ventricular arrhythmia in the FD-CIED group is shown in figure 2A, B. Of the 17 patients who had AF diagnosed on CIED follow-up, three strokes were recorded prior to anticoagulation, and no further episodes were documented after treatment. There were no differences in the incidence of arrhythmia needing therapy between those on ERT compared with those not on any disease-modifying therapy (online supplementary figure 1). Of the six FD-CIED patients with flow-limiting IHD, two were found to have an arrhythmia requiring medical treatment—one with NSVT and another with a short burst of AF (both less than 30 s in duration). There were 11 deaths during follow-up: two patients died following sustained ventricular arrhythmia, one patient with end-stage heart failure and the remainder died of non-cardiac causes.

10.1136/heartjnl-2019-315229.supp2 Supplementary data

From the FD-CIED cohort, 50 had defibrillator capability within their cardiac device (43 with an ICD and 7 with a cardiac resynchronisation therapy defibrillator (CRT-D). Sustained ventricular tachycardia (VT) needing anti-tachycardia pacing (ATP) or defibrillation occurred in 28% of patients (14/50) over a median follow-up duration of 3.7 years (IQR 1.8–6.2 years). VT with haemodynamic compromise needing defibrillation occurred in 16% of FD-CIED patients (8/50). Figure 2C shows a Kaplan-Meier curve for the occurrence of an arrhythmic event needing ICD therapies (ATP and defibrillation) in this cohort. Inappropriate defibrillation occurred in 2/50 patients (4%), both of whom subsequently underwent lead repositioning. When comparing those who had class 1 indications for ICD insertion to those who had a device implanted for primary prevention outside of guidelines, there were no differences in the occurrence of all arrhythmia types (any arrhythmia requiring therapy: 6/12 vs 8/19, p=0.410; VT with haemodynamic compromise requiring defibrillation: 3/12 vs 0/19, p=0.087; AF: 2/12 vs 2/19, p=0.277).

#### Arrhythmia follow-up: FD-control group

Of the FD-control cohort, 85/276 (31%) underwent Holter monitoring during the study period, with 12 of these identifying abnormalities. An alteration in therapy was required in 10/85 patients (12%): NSVT: four requiring medication and one currently under consideration for ICD implantation; SVT: four requiring anticoagulation/beta blockade for AF and one medical treatment for AV nodal re-entrant tachycardia). There were four deaths during follow-up: one sudden cardiac death suspected to be arrhythmic in nature and the remaining three from non-cardiac causes.

#### Clinical characteristics: FD-CIED versus FD-control

Patients with a cardiac device were older and more often male compared with those in the control cohort (table 2). There were also differences in electrical and structural parameters on baseline cardiac investigations, with the FD-CIED group having a longer PR interval and QRS duration, a greater LV mass, more scar tissue and more frequent LA dilatation. The distribution of baseline characteristics in different event categories (NSVT, AF and sustained VT/VF needing defibrillation) can be seen in table 5. There were no differences in clinical, ECG or imaging parameters between FD-CIED patients with arrhythmia requiring therapy (NSVT, AF and sustained VT/VF) compared with those who did not have arrhythmia. In the FD-control cohort, however, there was a tendency towards a higher LV mass, dilated left atrium, prolonged PR interval and QRS duration and an elevated MSSI in those who had an arrhythmia requiring treatment, but this did not reach statistical significance as we believe the number of events was low. Analysis using Cox regression showed no significant predictors for the presence of any arrhythmia requiring treatment (online Supplementary Table 1). In addition to the eight patients who had haemodynamically significant VT needing defibrillation, one further patient with a PPM died from sustained VT and VF identified from postmortem device interrogation. Of these nine patients with symptomatic VT, seven shared the following clinical characteristics: male >40 years, severe LVH, extensive LGE and an abnormal ECG with a QRS duration >120 ms. The remaining two patients included a female with coexisting familial long QT syndrome and a history of recurrent sustained VT and a 52 year-old man who had an ICD implanted following a short episode of NSVT.

**Table 5:** Distribution of baseline characteristics according to outcome: treated arrhythmia versus no arrhythmia

	FD-CIED			FD-control		
A. NSVT						
	No NSVT (n=49)	NSVT (n=41)	P value	No NSVT (n=221)	NSVT (n=5)	P value
LVH (n, %)	41 (83.7)	37 (90.2)	*0.353*	32/86 (37.2)	4/5 (80)	*0.078*
LGE* (n, %)	14/21 (66.7)	15/19 (78.9)	*0.648*	83/207 (40.1)	2/3 (66.7)	*0.567*
LA dilatation* (n, %)	28 (57.1)	28 (68.3)	*0.815*	12/57 (21.1)	1/4 (25)	*NS*
PR interval (ms)	186±42	163±36	***0.020***	145±28	157±35	*0.455*
QRS duration (ms)	138±34	133±30	*0.520*	99±20	118±32	*0.257*
MSSI	15.4±10.6	14.7±8.5	*0.742*	6.6±6.9	16.6±12.3	***0.013***
B. AF						
	No AF (n=73)	AF (n=17)	P value	No AF (n=222)	AF (n=4)	P value
LVH (n, %)	61 (83.5)	17 (100)	*0.575*	32/87 (36.8)	4/4 (100)	***0.022***
LGE* (n, %)	19/29 (65.5)	10/11 (90.9)	***0.033***	84/207 (40.6)	1/3 (33.3)	*NS*
LA dilatation* (n, %)	43 (58.9)	13 (76.4)	*0.562*	11/58^19^	2/3 (66.7)	*0.112*
PR interval (ms)	172±39	182±46	*0.440*	147±27	218	***0.011***
QRS duration (ms)	135±33	139±30	*0.686*	99±20	123±12	***0.041***
MSSI	15.4±10.4	13.7±5.9	*0.504*	7.1±7.7	12±7.2	*0.110*
C. VT with haemodynamic compromise or VF needing defibrillation						
	No ICD shock (n=82)	ICD shock (n=8)	P value			
LVH (n, %)	72 (87.8)	6 (75)	***0.046***			
LGE* (n, %)	26/37 (70.2)	3/3 (100)	*0.542*			
LA dilatation* (n, %)	52 (63.4)	4 (50)	*0.256*			
PR interval (ms)	175±41	166±37	*0.526*			
QRS duration (ms)	138±32	113±18	*0.071*			
MSSI	15.2±9.3	13.1±13.9	*0.586*			

The presence of specific clinical characteristics was evaluated in those with a treated arrhythmia in the FD-CIED and FD-control cohorts: NSVT (table 5a), AF (table 5b) and VT requiring defibrillation (table 5c, FD-CIED group only). Arrhythmia data were collected from CIED follow-up in the FD-CIED group and from Holter monitor testing in the FD-control group.

*Not all underwent CMR or transthoracic echocardiography imaging.

AF, atrial fibrillation; CMR, cardiovascular magnetic resonance; FD-CIED, Fabry disease with a cardiovascular implantable electronic device; ICD, implantable cardioverter defibrillator; LA, left atrium; LGE, late gadolinium enhancement; LVH, left ventricular hypertrophy; MSSI, Mainz Severity Score Index; NS, non-significant; NSVT, non-sustained ventricular tachycardia; VF, ventricular fibrillation; VT, ventricular tachycardia.

## Discussion

This study has shown that device implantation is frequent despite a lack of disease-specific guidance in FD.^8 23^ The burden of arrhythmia detected following device implantation was high, and subsequent modification of therapy was common. ICD-delivered therapy (ATP and defibrillation) occurred in over a quarter of the CIED population, highlighting the increased incidence of life-threatening arrhythmia in these patients. No significant differences were seen between types of CIEDs, suggesting that electrical changes appear to represent progressive cardiac disease in all FD patients. Diagnosis of asymptomatic AF from a cardiac device was frequent, and it is possible that this may contribute to the increased risk of ischaemic stroke, the second highest cause of mortality in FD patients. Although the indication for ICD implantation in FD was variable, an increasing number are inserted outside of guidance for primary prevention. Of note, almost a third of patients had a device inserted before a diagnosis of FD was made.

Arrhythmia is a common cause of morbidity and mortality in FD, yet there is no consensus evidence to guide treatment in FD patients, and the frequency of VT and VF reported in the literature varies widely.^8–11^ Previous studies have reported very low levels of clinically significant arrhythmia identified on device follow-up with a ventricular arrhythmia incidence of 5.2% over 4 years^9^ and atrioventricular conduction disease of 3.8% over 2.8 years.^24^ In our multicentre population, however, the burden of ventricular arrhythmia was much higher, with 28% needing device therapy over a 4-year follow-up period. Although the precise mechanisms of arrhythmia are not fully understood, certain structural changes may predispose to ventricular arrhythmia and sudden cardiac death, including LVH, ventricular dysfunction and extensive fibrosis with myocardial scarring.^25^ The evidence of an underlying inflammatory component leading to scar may be a potential explanation for any increase in arrhythmic risk in FD patients with LGE on CMR, but further research is needed.^26^

Ischaemic stroke is a common and serious clinical finding in early and advanced FD, with vascular endothelial sphingolipid accumulation thought to play a predominant role in its aetiology.^27^ Our study has shown that the rate of asymptomatic paroxysmal AF in those with advanced FD cardiomyopathy is high, suggesting that thromboembolic disease may have a more significant role in the aetiology of ischaemic stroke. In our control cohort, the rate of Holter monitoring in clinical practice was low, since monitoring was not a part of the Fabry400 protocol, suggesting that a greater burden of AF may be detected with more detailed surveillance. There is currently no FD-specific guidance for choice of anticoagulant therapy, and the use of the CHA_2_DS_2_-VASc scoring system is not recommended in FD to guide its initiation.^28 29^ Given the high incidence and risk of stroke in FD, current literature suggests lifelong anticoagulation in those with AF.^28^

Limited data have identified the following potential arrhythmic risk factors in FD: LVH, the presence of LGE on CMR, left atrial dilatation, a QRS duration greater than 120 ms, previously documented NSVT and an elevated MSSI above 20.^13^ Additionally, ECG abnormalities, including a prolonged PR interval and QRS duration, have been identified as independent predictors of pacemaker implantation in FD.^14^ These factors are common in FD patients who have a cardiac device but are also common in a large proportion of FD patients without a cardiac device.^30^ Male patients tend to have progressive disease with extensive cardiac involvement, and in combination with advancing age, these factors are thought to increase risk of ventricular arrhythmia.^13^ Further prospective research is needed to clarify risk factors on which recommendations for device implantation could be based. It is of great concern that almost a third of devices were implanted before a diagnosis of FD was confirmed, demonstrating that delay in diagnosing FD remains an issue in real-world practice.

The limitations of this study include the possibility that the burden of arrhythmia could be underestimated. Specific arrhythmias, such as slow VT not within the device detection range, may have been overlooked. Additionally, there may have been inappropriate identification of arrhythmia by the device, such as AF or artefact mistaken for VT, and consequent mislabeling of a rhythm disturbance. To minimise this, in cases where diagnosis of arrhythmia was unclear from the device interrogation report, all ECG traces were reviewed. Furthermore, a large proportion of Holter monitors were performed within local hospitals and data were not fully available in specialist centres. Consequently, it was not feasible to exclude arrhythmic events in the entire FD-control cohort, and definitive comparisons cannot be made of arrhythmic burden or presence of risk factors between the control and CIED groups. Further prospective studies are needed to definitively characterise arrhythmic risk and guide treatment based on their risk stratification.

## Conclusion

Cardiac device implantation is variable and does not always follow current guidance. This is true particularly for ICDs with many devices now inserted based on clinical suspicion of arrhythmia. Arrhythmias are common in Fabry patients following CIED implantation, with high rates of asymptomatic AF requiring anticoagulation and ventricular arrhythmia needing ICD device treatment. These are as high as those seen in HCM, supporting the need for prospective studies to inform Fabry-specific guidance for cardiac device implantation.

**Key messages**

**What is already known on this subject?**

- Cardiac involvement in Fabry disease is characterised by progressive left ventricular hypertrophy, myocardial fibrosis and arrhythmia. The incidence of atrial and ventricular arrhythmia reported in the literature is variable. Although some clinical and imaging parameters have been described as markers of arrhythmia, no definitive criteria exist to guide device implantation.

**What might this study add?**

- This is the first multicentre study evaluating cardiac device implantation practice, arrhythmia burden and device usage in Fabry disease. This study demonstrates that indications for implantable cardioverter-defibrillator (ICD) implantation are variable, and devices are often inserted for primary prevention outside of current guidance. The occurrence of asymptomatic AF and ventricular arrhythmia requiring ICD therapy is high in those with a cardiac device, demonstrating an increased arrhythmic risk in those with advanced cardiac disease.

**How might this impact on clinical practice?**

- Asymptomatic atrial fibrillation needing anticoagulation and ventricular arrhythmia requiring ICD therapy were as common as that observed in hypertrophic cardiomyopathy, supporting the need for Fabry-specific guidance for medical therapy and device implantation.
