# Supplementary material for: Study of indications for cardiac device implantation and utilisation in Fabry cardiomyopathy
Source: Heart. 2019 Aug 24;105(23):1825–31. doi: 10.1136/heartjnl-2019-315229 (PMC6900228; doi:10.1136/heartjnl-2019-315229)
Supplement: Supplementary data [file heartjnl-2019-315229supp001.pdf]

Supplementary Table 1. The relationship of baseline clinical characteristics and occurrence of any arrhythmia requiring treatment during follow-up (Cox regression analysis)

**Predictor variable**		**Multivariable analysis**		
		**HR**	**95% CI**	***p-value***
Age (years)	41-50	0.44	0.10-2.03	*0.292*
	51-60	0.77	0.23-2.55	*0.663*
	61-70	0.69	0.18-2.67	*0.591*
	>70	1.73	0.43-6.93	*0.438*
Male sex		0.76	0.30-1.92	*0.568*
QRSd (ms)*		0.93	0.83-1.06	*0.270*
LGE		2.12	0.56-8.05	*0.270*

*HR for QRSd is defined using 10ms intervals.

Table legend: CI – confidence interval, HR – hazard ratio, LGE – late gadolinium enhancement, QRSd – QRS duration.

Supplementary table 2. Implantable Loop Recorder (ILR) usage in Fabry disease for the duration of the study period.

	**Male**	**Female**	***p-value***
Sample size (n, %)	10	9	*N/A*
Age (years)	46±5	58±13	***0.023***
Follow-up (years)	2.2 (1.5-4.6)	1.7 (0.8-3.5)	*0.497*
**Indication:**			
Palpitations & dizziness (n, %)	6	2	*0.139**
Previous stroke ?AF (n, %)	2	1	
Suspected VA (n, %)	2	3	
Inconclusive Holter (n, %)	0	3	
**Arrhythmia:**			
AF requiring anticoagulation (n, %)	2	1	*0.498**
High VE burden (n, %)	1	0	
Bradycardia/pauses – did not fulfil criteria for PPM (n, %)	1	3	

P-values are for male-female comparisons. *statistical significance investigated using Chi-squared testing.

Table legend: AF – atrial fibrillation, PPM – permanent pacemaker, VE – ventricular arrhythmia

Supplementary table 3. Baseline demographic data in the FD-CIED cohort split by device type

	**PPM**	**ICD**	**CRT**	***p-value****
Sample size (n)	38	43	9	*N/A*
Age (yrs)	53±13	57±12	63±9	*0.052*
Male gender (n, %)	27 (71)	33 (77)	9 (100)	*0.182*
On ERT (n, %)	27 (71)	31 (72)	4 (44)	*0.247*
Classical mutation (n, %)	17 (45)	17 (40)	5 (56)	*0.660*
BMI (kg/m^2)	26±5	29±6	21±7	***0.014***
SBP (mmHg)	126±15	122±20	136±31	*0.499*
DBP (mmHg)	75±8	70±12	86±21	*0.077*
MSSI	14.5±10.7	14.8±8.6	18.4±10	*0.541*
**Comorbidities**				
IHD (n, %)	3 (8)	2 (5)	1 (11)	*0.719*
CKD stage 3-5 (n, %)	10 (26)	5 (12)	3 (33)	*0.147*
HTN (n, %)	8 (21)	8 (19)	2 (22)	*0.948*
DM (n, %)	3 (8)	5 (12)	0 (0)	*0.516*
Stroke/TIA (n, %)	14 (37)	5 (12)	3 (33)	***0.025***

*p-values are calculated using one-way ANOVA with post-hoc Tukey correction for continuous variables and Chi-squared testing for categorical variables.

Table legend: BMI – body mass index, CKD – chronic kidney disease, CRT – cardiac resynchronization therapy, DBP – diastolic blood pressure, DM – diabetes mellitus, ERT – enzyme replacement therapy, HTN – hypertension, ICD – implantable cardioverter defibrillator, IHD – ischemic heart disease, MSSI – Mainz severity score index, PPM – permanent pacemaker, SBP – systolic blood pressure, TIA – transient ischemic attack.

Supplementary figure 1

The incidence of arrhythmic events in the FD-CIED group on ERT vs. no therapy. It includes the following: (i) total documented arrhythmia, (ii) treated arrhythmia (including initiation of or a change in medication) and (iii) individual arrhythmia (AF and sustained VT requiring therapy). * denotes p=0.008. All other comparisons were non-significant.

Figure legend: AF – atrial fibrillation, ATP – anti-tachycardia pacing, VT – ventricular tachycardia.
