# Supplementary material for: Study of indications for cardiac device implantation and utilisation in Fabry cardiomyopathy
Source: Heart. 2019 Aug 24;105(23):1825–31. doi: 10.1136/heartjnl-2019-315229 (PMC6900228; doi:10.1136/heartjnl-2019-315229)

Supplementary figure 1. Incidence of arrhythmic events in those on ERT vs. no therapy (FD cohort with cardiac devices)

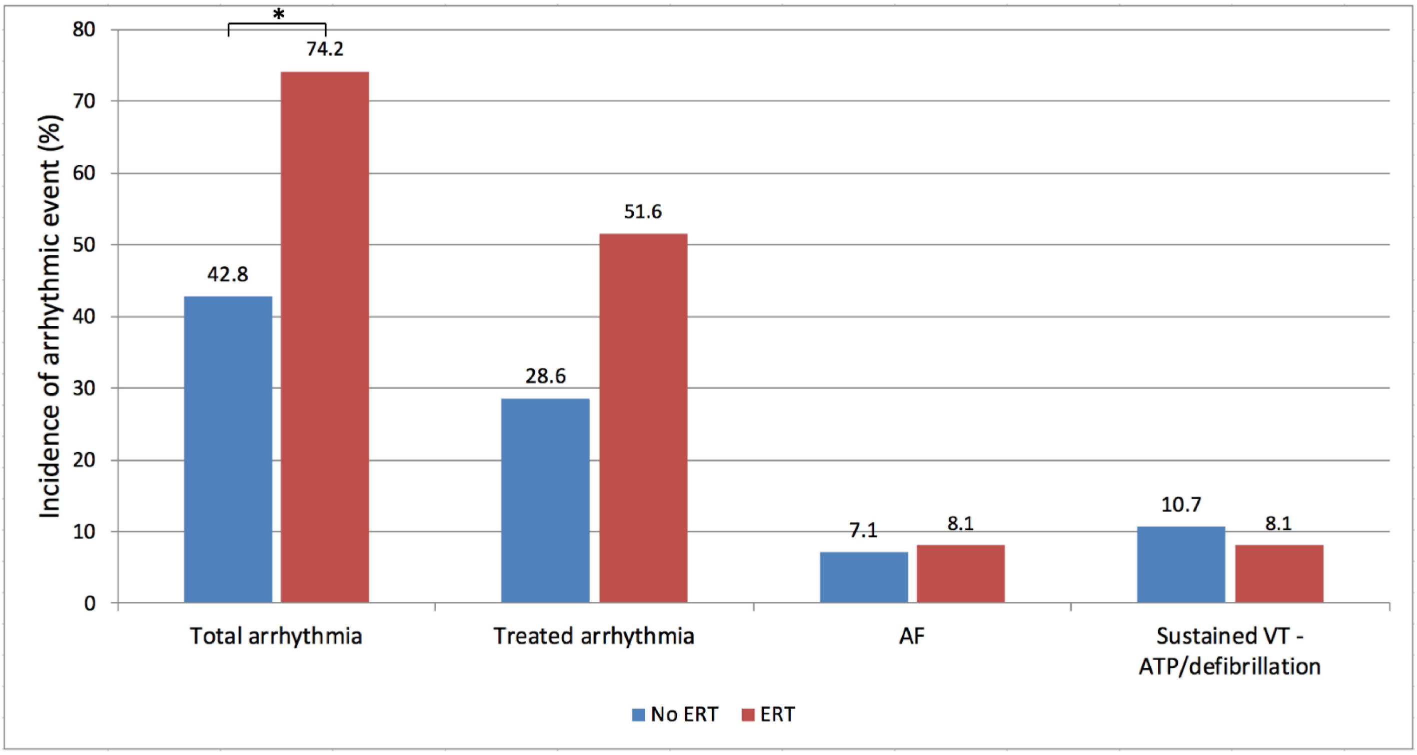

Supplement: Supplementary data [file heartjnl-2019-315229supp002.pdf]
